# Mutagenesis and expression of methane monooxygenase to alter regioselectivity with aromatic substrates

**DOI:** 10.1093/femsle/fnx137

**Published:** 2017-06-30

**Authors:** Malcolm Lock, Tim Nichol, J. Colin Murrell, Thomas J. Smith

**Affiliations:** 1 Biomolecular Sciences Research Centre, Sheffield Hallam University, Sheffield S1 1WB, UK; 2 School of Environmental Sciences, University of East Anglia, Norwich NR4 7TJ, UK^.^

**Keywords:** protein engineering, hydrocarbon oxidation, biocatalysis, methane, monooxygenase

## Abstract

Soluble methane monooxygenase (sMMO) from methane-oxidising bacteria can oxygenate more than 100 hydrocarbons and is one of the most catalytically versatile biological oxidation catalysts. Expression of recombinant sMMO has to date not been achieved in *Escherichia coli* and so an alternative expression system must be used to manipulate it genetically. Here we report substantial improvements to the previously described system for mutagenesis of sMMO and expression of recombinant enzymes in a methanotroph (*Methylosinus trichosporium* OB3b) expression system. This system has been utilised to make a number of new mutants and to engineer sMMO to increase its catalytic precision with a specific substrate whilst increasing activity by up to 6-fold. These results are the first ‘proof-of-principle’ experiments illustrating the feasibility of developing sMMO-derived catalysts for diverse applications.

## Teaser

Regioselectivity of soluble methane monooxygenase towards an aromatic substrate was increased via site-directed mutagenesis, indicating that this broad substrate-range enzyme can be engineered into a precise biocatalyst.

## INTRODUCTION

**Figure 1.**
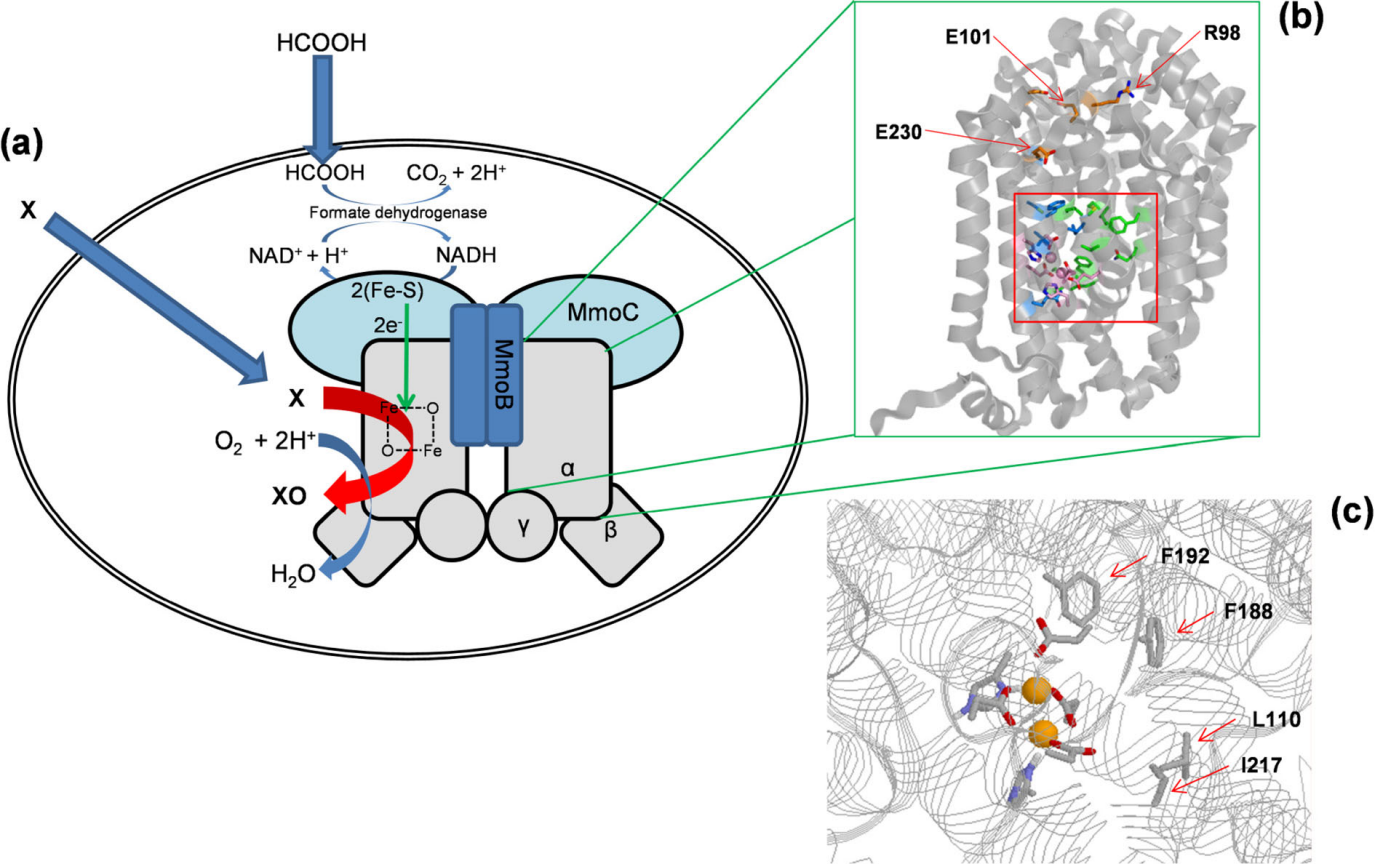
(**a**) Components of sMMO illustrating how the recombinant (and wild-type) whole-cell biocatalysts allow oxygenation of methane and other substrates (X converted to XO), driven by O_2_ and electrons supplied from externally supplied formate via formate dehydrogenase. The three subunits of the hydroxylase component of sMMO are shown in grey. The reductase component (MmoC—supplies electrons from NADH) is shown in turquoise and protein B (MmoB—also needed for full activity) in dark blue. (**b**) α Subunit of the *M. trichosporium* sMMO hydroxylase (constructed using crystallographic data from Elango *et al.* (1997)) showing the active centre (iron atoms as pink spheres; iron ligands in pink; residues lining the hydrophobic pocket in green) and representative residues of each of the three ionic networks (brown) around the proposed pathway of ingress of substrates into the active site. (**c**) Expanded view of the active centre showing the mutated residues F192 and I217, together with L110 mutated previously and F188. F188 is spatially close to F192 mentioned in the text.

Soluble methane monooxygenase (sMMO) is one of two enzyme systems in methane-oxidising bacteria (methanotrophs) that are able to perform the chemically challenging reaction of oxidising methane under ambient conditions using O_2_ as the oxidant. sMMO is multicomponent enzyme (Fig. 1a) in which oxygenation reactions occur at a diiron centre in the α-subunit (MmoX) of the hydroxylase component. A reductase (MmoC) provides electrons required by the reaction under the mediation of a third component (MmoB) (Hanson and Hanson 1996; Hakemian and Rosenzweig 2007; Smith and Murrell 2009). The exceptionally wide substrate range of sMMO, including many hydrocarbons and chlorinated hydrocarbons (Lipscomb 1994; Smith and Dalton 2004), has attracted the attention of biotechnologists for environmentally friendly catalysis of hydroxylation and epoxidation reactions despite the low regioselectivity and enantioselectivity of the wild-type enzyme. New catalysts for regiospecific oxygenation of unfunctionalised C-H bonds are in demand because there is a lack of workable catalytic technology of any kind, chemical or biological, to perform such transformations (Lewis, Coelho and Arnold 2011). Numerous diverse oxygenase enzymes can perform such reactions using dioxygen as the oxidant. Such enzymes potentially offer environmentally friendly routes to specific functionalisation with low cost and high atom efficiency (i.e. low wastage of starting materials). Excellent progress has been made with a number of specific reactions, notably cytochromes P450, for regioselective oxygenation of complex molecules, such as stereoselective hydroxylation at the 11-position of 11-deoxycortisol for commercial preparation of hydrocortisone (Li *et al.*^2002^; Julsing *et al.*^2008^). There remains, however, a lack of generally applicable technology for specific oxygenation reactions in largely unfunctionalised organic molecules.

sMMO belongs to a class of bacterial oxygenases referred to as soluble diiron monooxygenases (SDIMOs). These enzymes have been explored as practical oxidation catalysts since different members of the group oxygenate a wide range of aromatic and aliphatic hydrocarbons (Leahy, Batchelor and Morcomb 2003; Coleman, Bui and Holmes 2006), and manipulation of their catalytic properties via protein engineering is an attractive possibility. SDIMOs are complex enzymes of at least three components comprising up to six different polypeptide subunits and three to four redox active centres. All SDIMOs studied to date have proven challenging in terms of construction and functional expression of mutants, particularly those that naturally oxidise gaseous substrates (Canada *et al.*^2002^; Leahy, Batchelor and Morcomb 2003; Coleman, Bui and Holmes 2006; Feingersch *et al.*^2008^). There are a number of key SDIMOs that have not been expressed in active form in *Escherichia coli* despite considerable effort (Smith *et al.*^1999^; Chion, Askew and Leak 2005). There may be problems with engineering the expression of all required genes from bacteria distantly related to the host and possibly also with assembly of the terminal oxygenase complex and its diiron site. Among these, sMMO, although it offers considerable opportunities due to its broad substrate range and role in biological methane oxidation, has proven particularly challenging to achieve heterologous expression. In order to address these challenges, we and others have developed expression systems using alternative (and generally less genetically tractable) expression hosts (Jahng *et al.*^1996^; Lloyd *et al.*^1999^). For all mutagenesis experiments, we have favoured a homologous expression system where sMMO is expressed in an sMMO-deleted methane-oxidising bacterium, i.e. in the cellular milieu where the enzyme is naturally expressed (Smith *et al.*^2002^; Borodina *et al.*^2007^). This system, based on the well-characterised methane-oxidising bacterium *Methylosinus trichosporium* OB3b, although considerably more difficult to work with than *E. coli* and other well-developed expression hosts, allows recombinant genes to be introduced into the host by means of conjugation and gives sMMO expression levels indistinguishable from the wild-type methanotroph. When induced naturally by low-copper-to-biomass ratio, recombinant wild-type sMMO is produced in soluble extracts with activities of around 260 nmol min^−1^ mg of protein^−1^ oxygenation product with the standard assay substrate propene. This system can be used even to express completely inactive mutants of sMMO during growth on methane (Smith *et al.*^2002^) because *M. trichosporium* OB3b expresses the copper-containing membrane-bound particulate methane monooxygense (pMMO) when the copper-to-biomass ratio is sufficiently high (Hanson and Hanson 1996; Smith and Murrell 2009). Traces of the membrane-associated pMMO can either be removed by cell breakage and ultracentrifugation or, when transforming aromatic substrates (that are oxidised by sMMO but not pMMO), no cell fractionation is required.

Whilst available expression systems for sMMO have limitations in terms of the number of mutants that can be constructed and analysed, the homologous expression system has allowed mutagenesis and expression of a small number of active-site mutants that have given the first and to date only mutagenesis-based evidence for the roles of specific amino acyl residues in the function of this enzyme. In particular, previous mutagenesis results have shown that it is possible to influence the orientation of aromatic substrates in the active site of sMMO via mutations at Leu 110 (Borodina *et al.*^2007^; Fig. 1). Leu 110 is one of the residues lining the hydrophobic pocket in the α-subunit (MmoX) of the hydroxylase component of the enzyme that has also been proposed to have a substrate-gating function (Rosenzweig *et al.*^1997^; Lee *et al.*^2013^). These mutants were, however, not of interest from a biotechnological viewpoint because mutations at this position (to larger or smaller residues) made the enzyme less regioselective with aromatic substrates than the wild type. Here we describe further improvements to the homologous expression system for sMMO that reduce the number of cloning steps for construction of each mutant from three (Smith *et al.*^2002^) to one. We have used this system to construct mutations to explore three new sites within the hydroxylase component of sMMO and report an increase in regioselectivity and specific activity for the aromatic substrate biphenyl.

## MATERIALS AND METHODS

### Expression system for sMMO

**Figure 2.**
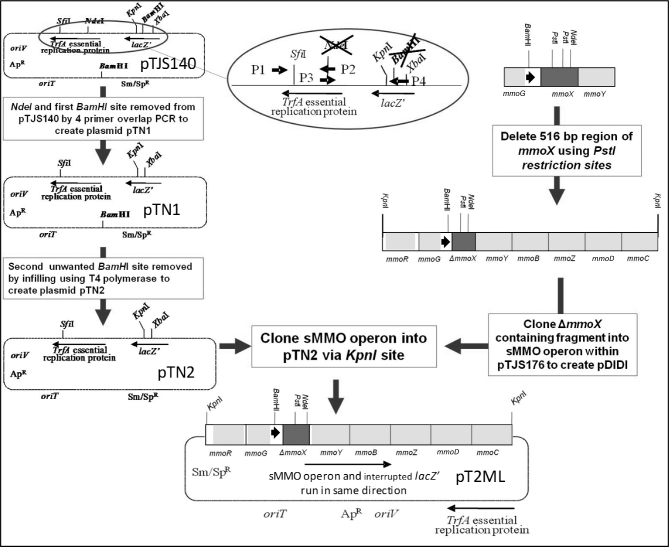
Construction of cloning vector pT2ML. The stout arrow within the sMMO-encoding operon indicates the approximate location of the natural promoter that directs expression of the sMMO structural genes at low copper-to-biomass ratio. In order to create the plasmids for expression of the recombinant sMMO variants, the internally deleted *ΔmmoX* (shaded in dark grey) was replaced by the full-length mutant (or wild-type) *mmoX* gene fragment via cloning with *Bam*HI and *Nde*I.

Recombinant sMMO was expressed in *Methylosinus trichosporium* SMDM, a strain with the six-gene operon encoding sMMO largely deleted (Borodina *et al.*^2007^). Plasmid pT2ML, a modified and improved version of pTJS175 that was used previously for mutagenesis of sMMO (Smith *et al.*^2002^; Borodina *et al.*^2007^), was engineered to allow cloning of mutants in a single step and to minimise unwanted wild-type clones, as indicated in Fig. 2 and described in greater detail in the Supporting Information.

The resulting plasmid, pT2ML, is an *E. coli*-*M. trichosporium* shuttle vector that permits cloning of mutated *mmoX* genes in a single directed cloning step using *Bam*HI and *Nde*I. (Fig. 2). Moreover, since the *mmoX* gene in pT2ML is inactivated by partial deletion, the presence of sMMO activity in the progeny or a full-size *mmoX* gene (detectable via PCR) shows cloning of a recombinant rather than religation of the parental plasmid.

pT2ML permits cloning of mutagenised fragments of the active site-encoding *mmoX*-gene in a single operation (using *Bam*HI and *Nde*I), and the presence of a deletion within the copy of *mmoX* in pT2ML allows easy screening of recombinants for the presence of the recombinant sMMO genes on the basis of their ability to oxidise naphthalene in a colorimetric spot test (Bodrossy *et al.*^1995^) as long as the recombinants, like the wild type, are active with this substrate. The recombinant sMMO genes are expressed from their natural promoters and Shine-Dalgarno sequences.

### Design of site-directed mutants

F192 (Fig. 1) was chosen as a site for mutagenesis because it is positioned at the same end of the substrate-binding pocket as the diiron centre and, together with another phenylalanyl residue (F188), interacts with an acetate ion in the structure of sMMO from *Methylococcus capsulatus* (Bath) (Rosenzweig *et al.*^1995^). Both phenylalanyl residues are conserved in the sMMO from *M. trichosporium* OB3b that was used here (Fig. 1c; Fig. S1, Supporting Information) and may be critical in determining the hydrophobic landscape next to the diiron centre where substrates bind. The mutation F192I was designed based on sequence alignments, where this position is occupied by Ile 179 in the homologous toluene 4-monooxygenase (T4MO) (Leahy, Batchelor and Morcomb 2003) that naturally oxygenates monoaromatic hydrocarbons. A natural variant was chosen to give a change that the structure is likely to be able to accommodate (Fig. S1). I217 is within the substrate binding pocket (Fig. 1c), though distal to the diiron centre and was converted to the less bulky alanyl residue, with the initial intention of producing a mutant that might (unlike the wild type) be able to accommodate and oxygenate triaromatic hydrocarbons in its active site. Three ionic networks near to the upper apex of the αβγ protomer of the sMMO hydroxylase (highlighted in different colours in the alignment in Fig. S1) were identified as possible mutagenesis sites that could be used to manipulate the conformational flexibility of the enzyme. It was also reasoned that mutations at these positions might modulate substrate access into the active centre, via the chain of internal cavities in this region within the protein that has been experimentally tracked by xenon gas in crystals of the homologous sMMO of *Mc. capsulatus* (Bath) (Whittington *et al.*^2001^). It is also known that this aperture is opened by binding of protein B to the hydroxylase (Lee *et al.*^2013^; Wang and Lippard 2014). In the *M. trichosporium* OB3b enzyme studied here, these networks comprise the R98/D365 ion pair, the D164/E101/K104/R360 network and ion pair between E230 and R12 (of the β-subunit) (the positions of these arginyl residues are indicated in Fig. 1b; and the residues participating in the three networks are shown in yellow, red and green, respectively, in Fig. S1). Here, the role of the R98/D365 ion pair was tested via the mutation R98L.

### Additional experimental details

Methods for site-directed mutagenesis, whole-cell biotransformation reactions, analysis of sMMO-expressing cells and molecular modelling are given in the Supporting Material.

## RESULTS

### Development of the expression system

In the previous form of the homologous expression system for sMMO (Smith *et al.*^2002^), a three-step cloning process was required in order to construct mutants of the MMO-encoding genes within the expression plasmid for *Methylosinus trichosporium* OB3b. Here, the expression system was streamlined to remove three restriction sites in order to allow cloning of mutant *mmoX* fragments (984 bp, permitting mutagenesis of amino acids 1–245 of the 526-amino-acid MmoX) prepared by PCR mutagenesis into the sMMO-encoding operon as a single directed cloning step, as described in the Materials and Methods section. This region includes 17 out of 19 active-site residues likely to interact with the substrate, as well as five out of six of the amino acids that ligate the diiron centre (Elango *et al.*^1997^). Also, a 516-bp deletion within the *mmoX* gene of the vector allows recombinants to be identified by the increase in size of a gene-specific PCR product and ensures that any observed sMMO activity must be due to the recombinant *mmoX* being cloned. Where a full-length mutant *mmoX* is cloned, the operon is expressed from its natural promoters and Shine-Dalgarno sequences, and (as in the parental methanotroph) is induced by low copper-to-biomass ratio.

### Mutagenesis and expression of mutant enzymes

The three mutants R98L, F192I and I217A were constructed in *Escherichia coli* in the new expression plasmid pT2ML, before conjugation into the sMMO-minus methanotroph strain *M. trichosporium* SMDM and expression of the mutant enzymes as described in the Materials and Methods section. Methanotroph cells expressing the wild-type sMMO in the same system were prepared for use as the wild-type control.

### Catalytic properties of the mutants

Whilst no activity could be detected with the triaromatic substrates anthracene and phenanthrene (data not shown), transformations using mono- and di-aromatic substrates (Table 1) revealed a number of changes in the properties of the enzyme. With toluene as the substrate, the R98L and I217A mutants gave similar regioselectivities to the wild type, namely a mixture of products with oxygenation on the sidechain and at the 4-position, i.e. at the ‘ends’ of the molecule if the substrate is considered to be a rectangle. The F192I mutant showed a substantial shift in regioselectivity towards the sidechain (*P* = 0.000, ANOVA test). With the bulkier substrate biphenyl, where hydroxylation by the wild-type sMMO was detectable at the 2- and 4-positions of the molecule, the F192I and I217A mutants showed the appearance of detectable amounts of 3-hydroxybiphenyl not seen with the wild type. The R98L mutant showed a substantial increase in activity (6-fold compared to the wild type; t-test comparison of the R98L and wild-type activities, *P* = 0.05) but also a substantial increase in regioselectivity for the ‘end’ 4-position, giving almost 98% pure 4-hydroxybiphenyl as the product, compared with the wild type where the proportion of 4-hydroxybiphenyl was only 94%. Comparison of these data using the ANOVA test showed significant differences between the wild-type and the R98L mutants (*P* = 0.046), and the wild-type and the I217A mutant (*P* = 0.001).

**Table 1.** Product distribution of wild-type and mutant enzymes with (a) the mono-aromatic substrate toluene and (b) the di-aromatic substrate biphenyl.

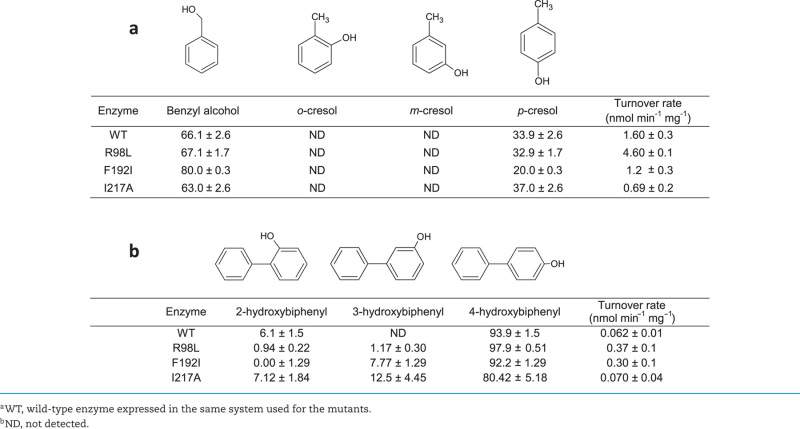									

## DISCUSSION

Progressive improvements have been made to the system for homologous expression of recombinant and mutant sMMO enzymes in a methanotroph host. Most recently (as reported here), the expression vector has been modified to allow cloning of mutant genes in a single step. This substantially improves practicability of the system, although further improvement (such as a promoter system independent of culture copper levels) would be desirable. Moreover, the use of a methane-oxidising bacterium as the expression host may be advantageous in offering the production of valuable biocatalysts using inexpensive methane as the feedstock, including fossil methane and biogas. The practicality of such a system on a large scale was shown by a pilot commercial biotransformation for production of racemic 1,2-epoxypropane from propylene using methanotroph cells that was conducted in the 1990s (Stanley and Dalton 1992; Richards *et al.*^1994^) and large-scale fermentation operations of methanotrophs to produce single-cell protein (Skrede *et al.*^1998^). The system developed here may also be useful for expressing other biotechnologically valuable proteins in methanotrophs using methane as the feedstock.

Other studies have indicated important residues in determining the catalytic properties of the enzyme in homologous enzyme systems, including increases in activity as shown in this report. In toluene/o-xylene monooxygenase (ToMO) and T4MO, mutations at the residue E214 (equivalent to A226 in sMMO and spatially near to R98 mutated in this study) around the mouth of the substrate-entry channel did not, unlike the R98L mutation in sMMO, change regioselectivity but influenced activity in the aromatic monooxygenases (Vardar and Wood 2005; Brouk, Nov and Fishman 2010). Notably, the E214G mutation in ToMO resulted in a 15-fold increase in oxygenation activity with the adventitious substrate 4-nitrophenol and, in combination with two other mutations, gives an activity enhancement of 20-fold relative to the wild type, resulting in a specific activity of 0.164 nmol min^−1^ mg^−1^ (Vardar and Wood 2005).

Other mutagenic studies of the SDIMOs that have resulted in increased activity and/or catalytic precision have included a study that increased activity of T4MO for synthesis of polyhydroxylated compounds including the food additive hydroxytyrosol (Brouk *et al.*^2010^; Brouk and Fishman 2012), increasing enantiomeric excess for the production of chiral sulfoxides (Feingersch *et al.*^2008^). Another study used directed evolution to produce an enzyme derived from toluene 2-monooxygenase whose improved properties included a 6.4-fold increase in naphthalene oxidation, to a specific activity of 0.19 nmol min^−1^ mg^−1^ (Canada *et al.*^2002^). Recently, mutations at F176 in ToMO (equivalent to F188 in sMMO) have been shown to increase activity by up to 4.7-fold and to give changes in regioselecitivty. These include the F176N mutant that gave greater direction towards the 4-position, most notably an increase of the proportion of *p*-cresol from oxidation of toluene from 51% in the wild type to 92% in the mutant, with a specific activity of 0.86 nmol min^−1^ mg^−1^ (54% of wild-type activity) (Sönmez *et al.*^2014^). Saturation mutagenesis of a number of sites in the active site, substrate access channel and elsewhere in ToMO also led to mutants with increases in rate and changes in regioselecitivty with monoaromatic substrates (Kurt *et al.*^2016^). There are reports in the literature of use of aromatic monooxygenases to produce tricyclic aromatic compounds as a result of addition reactions between oxygenate monoaromatic products (McClay *et al.*^2015^), but to our knowledge the situation across the SDIMOs as a whole is the same as that obtained here, that oxygenation of triaromatic substrates has yet to be achieved and may reflect the size limitation of the active site across the wild-type enzymes of the class. Here, we have shown that the highly versatile sMMO can be engineered to alter the regioselectivty with aromatic substrates, including more precise hydroxylation of biphenyl at the 4-position, and we have achieved what to our knowledge is the highest specific activity (0.37 nmol min^−1^ mg^−1^) towards so large a substrate by cells expressing this group of enzymes.

## Supplementary Material

Supplemental material Supplementary data are available at *FEMSLE* online.
