# Supplementary material for: Mutagenesis and expression of methane monooxygenase to alter regioselectivity with aromatic substrates
Source: FEMS Microbiol Lett. 2017 Jun 30;364(13):fnx137. doi: 10.1093/femsle/fnx137 (PMC5812538; doi:10.1093/femsle/fnx137)
Supplement: Supplemental material — Supplementary data are available at FEMSLE online. [file fnx137_supp.docx]

**Modification of the expression system for sMMO.** Plasmid pT2ML, a modified and improved version of pTJS175 that was used previously for mutagenesis of sMMO (Smith *et al.* 2002; Borodina *et al.* 2007), was engineered to allow cloning of mutants in a single step and to minimise unwanted wild-type clones, as detailed below and in Fig. 2. Two out of three unwanted restriction sites were removed from the *E. coli*-*M. trichosporium* shuttle vector pTJS140 (Smith *et al.* 2002) via a single operation of overlap extension PCR mutagenesis (Ho *et al.* 1989). The upstream and downstream external primers were, respectively, primer P1 (5’-CAC GCG AGG AAC TAT GAC GAC CAA GAA GC-3’) and P4 (5’- G TCG ACT CTA GAG GAC CCC CGG GTA CCG AG-3’). Primer P4 introduced a point mutation (bold) to remove the *Bam*HI (remnant underlined) site adjacent to the *Xba*I site (double underlined) that was used to clone the PCR product. The two internal PCR primers (P2, 5’-GTA CTT CTC CCA GAT GAA TTT CGT GTA G-3’, and its complement P3) introduced a point mutation (bold) to remove the *Nde*I site (remnant underlined) within the essential replication gene *trfA*, without altering the encoded amino acid sequence. The PCR product was introduced into pTJS140 as indicated in Fig. 2 and then the remaining *Bam*HI site was removed by cutting, in-filling and religation to give plasmid pTN2. A 513-bp *Pst*I fragment was excised from within *mmoX* in the sMMO-encoding operon; the operon was religated and cloned into pTN2 using *Kpn*I.

pT2ML (Fig. 2) and its predecessor expression plasmid pTJS175 contain the whole six-gene operon that encodes sMMO plus two genes upstream (*mmoR* and *mmoG*). It was necessary to include this relatively large (approximately 10 kb) segment of the *M. trichosporium* chromosome into the expression plasmid, even though all mutants were made within the *mmoX* gene, because it was observed previously (Smith *et al.* 2002) that a plasmid clone of the mmoX gene plus its promoter sequence did not restore functional sMMO expression in a strain of *M. trichosporium* where the chromosomal copy of mmoX was inactivated.

**Mutagenesis of sMMO.** Mutants were constructed via the four primer overlap-extension PCR method (Ho *et al.* 1989) as described previously (Smith *et al.* 2002) with primers detailed in Supplementary Table 1. Mutated PCR products were cloned into the remainder of the sMMO operon in pT2ML using *Bam*HI and *Nde*I. PCR-derived portions of all clones were verified free from unwanted mutations by dye termination sequencing.

Production of sMMO-containing whole-cell biocatalysts. pT2ML and its derivatives containing mutated sMMO genes were transferred from *E. coli* to *M. trichosporium* SMDM via conjugation, as described previously (Lloyd *et al.* 1999). The exconjugant *M. trichosporium* cells capable of expressing wild-type or mutant sMMO enzymes were grown in an atmosphere containing approximately 20 % (vol/vol) methane on NMS agar plates (Smith and Murrell 2011) containing 1 µg ml^-1^ of CuSO_4_.5H_2_O, together with gentamicin (5 µg ml^-1^), streptomycin (20 µg ml^-1^) and spectinomycin (20 µg ml^‑1^). Generally cultures of *Ms. trichosporium* OB3b initially grow using the copper-dependent pMMO and induce sMMO when as the copper-to-biomass ratio of the culture decreases. Therefore, the colorimetric naphthalene oxidation test (Bodrossy *et al.* 1995) was performed on a small amount of colony biomass removed from the plate to confirm that sMMO (the sole enzyme from *Ms. trichosporium* OB3b capable of oxidising naphthalene) was present. The cells were then washed off the plates into 25 mM MOPS buffer pH 7.0 to give a suspension of OD_600_ = 10 for use in the biotransformations. The aromatic substrates investigated during this study are not substrates for pMMO and so it was not necessary to test whether pMMO was expressed.

Laboratory scale biotransformations.

A 5 ml aliquot of the above bacterial suspension was incubated aerobically with shaking (200 rpm) with substrate (5 µl toluene for 5 min, 15 mg biphenyl for 1h, 15 mg phenanthrene or anthracene for 24 h) in the presence of 5 mM sodium formate to provide an excess of reducing equivalents for sMMO. Phenanthrene and anthracene Products were extracted and quantified via GC; details may be found in the supplemental material, together with sample traces showing resolution of the different products (Supplementary Figs. S2 and S3). Soluble protein concentrations were determined as indicated in the supplemental material. Protein profiles from SDS-PAGE and expression of the sMMO subunits were comparable in the wild-type and mutants used for biotranformations.

**GC detection, quantification of products and protein estimation**

The hydroxylated products were extracted from the 5-ml whole-cell biotransformation reactions with 1.25 ml of diethyl ether and evaporated down to 50 µl final volume. 2-Phenyl ethanol (100 nmoles) was used as the internal standard. Samples (1 µl) were analysed via gas chromatography (GC) using a GC-2010 (Shimadzu) coupled to a flame ionisation detector. The GC was fitted with a Restek RTX-5 column with a (5 % diphenyl, 95% dimethyl polysiloxane coating; 30 m × 0.32 mm; coating thickness 0.1 μm). When toluene was the substrate the GC was operated at a linear flow rate of 9.7 cm s^-1^ using N_2_ as the carrier gas and a 15:1 split. The column temperature was ramped from 80 ºC to 90 ºC at 0.5 ºC min^-1^ , held at 90 ºC for 1 min followed by ramp to 240 ºC at 25 ºC min^-1^ and held for 3 min. A representative GC trace is shown in Supplementary Fig. S2. When biphenyl was the substrate the GC was operated at a linear flow rate of 10.3 cm s^-1^ using N_2_ as the carrier gas with a 20:1 split. The column temperature held at 120 ºC for 3 min then ramped from 120 ºC to 180 ºC at 5 ºC min^-1^, held at 180 ºC for 1 min, followed by ramping to 250 ºC at 20 ºC min^-1^ and holding at for 3 mins. For all GC analyses the injection temperature was 300 ºC and the detector temperature was 250 ºC. Products were identified by comparison of retention times to authentic standards. Specific activities for each substrate were calculated as nmoles (product) min^-1^ mg (total soluble protein)^-1^. A representative GC trace is shown in Supplementary Fig. S3.

For soluble protein estimation, cells were broken by 3 passages through a French pressure cell (Thermo) at 137 MPa, centrifuged for 90 min (50,000 × *g*; 4 °C). Supernatant (100 µl) was added to 3 ml of Bradford protein assay reagent (Sigma) at room temperature and incubated for 5 min. Assays with the triaromatic compounds phenanthrene and anthracene did not produce detectable product peaks across a range of GC parameters. The increase in absorbance relative to a no-protein control was measured at 595 nm using a Jenway 6715 UV/Vis spectrophotometer and protein concentration estimated from comparison against a bovine serum albumin standard curve.

**Statistical analysis of data**

Statistical analysis was performed using the SPSS package (version 24, IBM Corporation). Percentage data were arcsine transformed before analysis. Data were tested for normality using the Shapiro-Wilk test and for homogeneity of variance using the Levene test. Paired sets of normally distributed data sets were tested for significance using the T-test and sets of normally distributed data that passed the homogeneity of variance test were tested for significance using a one-way ANOVA followed by the Tukey post-hoc test.

Molecular graphics. Molecular graphics were performed using Pymol (The PyMOL Molecular Graphics System, Version 1.5.0.4 Schrödinger, LLC).

TomA3 ------------MDTSVQKKKLGLKDRYAAMTRGLGWQTSYQPMEKVFPYDK--YEGIKI 46

PhN --------------MVSKNKKLNLKDKYQYLTRDMAWEPTYQDKKDIFPEED--FEGIKI 44

TouA -------------------MSMLKREDWYDLTRTTNWTPKYVTENELFPEEMSGARGISM 41

TmoA -------------------MAMHPRKDWYELTRATNWTPSYVTEEQLFPERMSGHMGIPL 41

OB3b-MmoX MAISLATKAATDALKVNRAPVGVEPQEVHKWLQSFNWDFK--ENRTKYPTKY-------- 50

McBath-MmoX MALSTATKAATDALAANRAPTSVNAQEVHRWLQSFNWDFK--NNRTKYATKY-------- 50

B276-AmoC --------------------MASNPTQLHEKSKSYDWDFTSVERRPKFETKY-------- 32

: * . . :

E101

**R98** K104

TomA3 HDWDKWEDPFRLTMDAYWKYQGEKEKKLYAVIDAFAQNNGQLSISDARYVNALKVFIQGV 106

PhN TDWSQWEDPFRLTMDAYWKYQAEKEKKLYAIFDAFAQNNGHQNISDARYVNALKLFISGI 104

TouA EAWEKYDEPYKITYPEYVSIQREKDSGAYSIKAALERD-GFVDRADPGWVSTMQLHFGAI 100

TmoA EKWESYDEPYKTSYPEYVSIQREKDAGAYSVKAALERA-KIYENSDPGWISTLKSHYGAI 100

OB3b-MmoX HMANETKEQFKVIAKEYARMEAAKDERQFGTLLDGLTRLGAGNKVHPRWGETMKVISNFL 110

McBath-MmoX KMANETKEQFKLIAKEYARMEAVKDERQFGSLQDALTRLNAGVRVHPKWNETMKVVSNFL 110

B276-AmoC KMPKKGKDPFRVLIRDYMKMEAEKDDRTHG-FLDGAVRTREATRIEPRFAEAMKIMVPQL 91

.. .: :: * : *: .. .. : .::: :

D164

TomA3 TPLEYMAHRGFAHIGRHFTGEGARVACQMQSIDELRHFQTEMHALSHYNKYF---NGLHN 163

PhN SPLEHAAFQGYSKVGRQFSGAGARVACQMQAIDELRHSQTQQHAMSHYNKHF---NGLHD 161

TouA ALEEYAASTAEARMARFAKAPGNRNMATFGMMDENRHGQIQLYFPYANVKRS---RKWDW 157

TmoA AVGEYAAVTGEGRMARFSKAPGNRNMATFGMMDELRHGQLQLFFPHEYCKKD---RQFDW 157

OB3b-MmoX EVGEYNAIAASAMLWDSATAAEQKNGYLAQVLDEIRHTHQCAFINHYYSKHYHDPAGHND 170

McBath-MmoX EVGEYNAIAATGMLWDSAQAAEQKNGYLAQVLDEIRHTHQCAYVNYYFAKNGQDPAGHND 170

B276-AmoC TNAEYQAVAGCGMIISAVENQELRQGYAAQMLDEVRHAQLEMTLRNYYAKHWCDPSGFDI 151

*: * . . : : :** ** : * .

F188

**F192** **I217** A226

TomA3 S-IHWYDRVWYLSVPKSFFEDAATG-GPFEFLTAVSFSFEYVLTNLLFVPFMSGAAYNGD 221

PhN G-PHMHDRVWYLSVPKSFFDDARSA-GPFEFLTAISFSFEYVLTNLLFVPFMSGAAYNGD 219

TouA AHKAIHTNEWAAIAARSFFDDMMMTRDSVAVSIMLTFAFETGFTNMQFLGLAADAAEAGD 217

TmoA AWRAYHSNEWAAIAAKHFFDDIITGRDAISVAIMLTFSFETGFTNMQFLGLAADAAEAGD 217

OB3b-MmoX ARRTRAIGPLWKGMKRVFADGFISG-DAVECSVNLQLVGEACFTNPLIVAVTEWASANGD 229

McBath-MmoX ARRTRTIGPLWKGMKRVFSDGFISG-DAVECSLNLQLVGEACFTNPLIVAVTEWAAANGD 229

B276-AmoC GQRGLYQHPAGLVSIGEFQH-FNTG-DPLDVIIDLNIVAETAFTNILLVATPQVAVANGD 209

. * . ... : : * :** :: * **

E320 F236

TomA3 MSTVTFGFSAQSDESRHMTLGIECIKFMLEQDPDNVPIVQRWIDKWFWRGYRLLSIVA-M 280

PhN MATVTFGFSAQSDEARHMTLGLEVIKFILEQHEDNVPIVQRWIDKWFWRGFRLLSLVS-M 278

TouA HTFASLISSIQTDESRHAQQGGPSLKILVENGKKDE--AQQMVDVAIWRSWKLFSVLTGP 275

TmoA YTFANLISSIQTDESRHAQQGGPALQLLIENGKREE--AQKKVDMAIWRAWRLFAVLTGP 275

OB3b-MmoX EITPTVFLSVETDELRHMANGYQTVVSIAND-PASAKFLNTDLNNAFWTQQKYFTPVLGY 288

McBath-MmoX EITPTVFLSIETDELRHMANGYQTVVSIAND-PASAKYLNTDLNNAFWTQQKYFTPVLGM 288

B276-AmoC NAMASVFLSIQSDEARHMANGYGSVMALLEN-EDNLPLLNQSLDRHFWRAHKALDNAVGW 268

.. * ::** ** * : : :: . : :: :* : :

TomA3 MQDYMLPNRVMSWR-ESWEMYVEQNGGALFKDLARYGIRKPKGWDQACEGKDHISHQTFA 339

PhN MMDYMLPNKVMSWS-EAWEVYYEQNGGALFKDLERYGIRPPKYQDVANDAKHHLSHQLWT 337

TouA IMDYYTPLESRNQ--SFKEFMLEWIVAQFERQLLDLGLDKPWYWDQFMQDLDETHHGMHL 333

TmoA VMDYYTPLEDRSQ--SFKEFMYEWIIGQFERSLIDLGLDKPWYWDLFLKDIDELHHSYHM 333

OB3b-MmoX LFEYGSKFKVEPWVKTWNRWVYEDWGGIWIGRLGKYGVESPASLRDAKRDAYWAHHDLAL 348

McBath-MmoX LFEYGSKFKVEPWVKTWDRWVYEDWGGIWIGRLGKYGVESPRSLKDAKQDAYWAHHDLYL 348

B276-AmoC CSEYGARKRPWSYKAQWEEWVVDDFVGGYIDRLSEFGVQAPACLGAAADEVKWSHHTLGQ 328

:* . . : . * *: * *

R360 D365

TomA3 VFYNYNAAAPIHTWVPTK-EEMGWLSEKYPETFDKYYRPRWDYWREQAAKGNR------- 391

PhN TFYQYCQATNFHTWIPEK-EEMDWMSEKYPDTFDKYYRPRYEYLAKEAAAGRR------- 389

TouA GVWYWRPTVWWDPAAGVSPEEREWLEEKYPG-WNDTWGQCWDVITDNLVNGKPE------ 386

TmoA GVLDWRTTAWWNPAAGVTPEERDWLEEKYPG-WNKRWGRCWDVITENVLNDRMD------ 386

OB3b-MmoX AAYAMWPLGFARLALPDE-EDQAWFEANYPG-WADHYGKIFNEWKKLGYEDPKSGFIPYQ 406

McBath-MmoX LAYALWPTGFFRLALPDQ-EEMEWFEANYPG-WYDHYGKIYEEWRARGCEDPSSGFIPLM 406

B276-AmoC VLSAVWPLNFWRSDAMGP-ADFEWFENHYPG-WSAAYQGYWEGYKAL--ADPAGGRIMLQ 384

: *:. :** : : :: .

TomA3 --FYNKTLPMLCTTCQIPMIFTEPGDATKICYRESAYLGDKYHFCSDHCKEIFDNEPEKF 449

PhN --FYNNTLPQLCQVCQIPTIFTEKDAPTMLSHRQIEHEGERYHFCSDGCCDIFKHEPEKY 447

TouA -LTVPETLPTICNMCNLPIAHTP-GNKWNVKDYQLEYEGRLYHFGSEADRWCFQIDPERY 444

TmoA -LVSPETLPSVCNMSQIPLVGVP-GDDWNIEVFSLEHNGRLYHFGSEVDRWVFQQDPVQY 444

OB3b-MmoX WLLANGHDVYIDRVSQVPFIPSL--AKGTGSLRVHEFNGKKHSLTDDWGERQWLIEPERY 464

McBath-MmoX WFIENNHPIYIDRVSQVPFCPSL--AKGASTLRVHEYNGEMHTFSDQWGERMWLAEPERY 464

B276-AmoC ELPG---LPPMCQVCQVPCVMPR--LDMN-AARIIEFEGQKIALCSEPCQRIFTNWPEAY 438

: .::* . * : .: : * :

TomA3 VQSWLPPQQVYQGNCFKPDADPTKEGFDPLMALLDYYNLNVGRDNFDFEGSEDQKNFAAW 509

PhN IQAWLPVHQIYQGNC---------EGGDLETVVQKYYHINIGEDNFDYVGSPDQKHWLSI 498

TouA KNHTNLVDRFLKGEIQP----ADLAGALMYMSLEP-GVMGDDAHDYEWVKAYQKKTNAA- 498

TmoA QNHMNIVDRFLAGQIQP----MTLEGALKYMGFQSIEEMGKDAHDFAWADKCKPAMKKSA 500

OB3b-MmoX ECHN-VFEQYEGRELSEVIAEGHGVRSDGKTLIAQPHTRGDNLWTLEDIKRAGCVFPDPL 523

McBath-MmoX ECQN-IFEQYEGRELSEVIAELHGLRSDGKTLIAQPHVRGDKLWTLDDIKRLNCVFKNPV 523

B276-AmoC RHRKQYWARYHGWDLADVIVDLGYIRPDGKTLIGQPLLEMERLWTIDDIRALQYEVKDPL 498

: : . .

TomA3 RGEVLQGEAK--- 519

PhN KGRKPADKNQDAA 511

TouA -------------

TmoA -------------

OB3b-MmoX AKF---------- 526

McBath-MmoX KAFN--------- 527

B276-AmoC QEA---------- 501

**Supplementary Figure S1.** Alignment of MmoX with representative homologous oxygenase subunits showing mutagenised residues and other sites mentioned in the text. Residues indicated above the alignment are numbered as in MmoX; residues mutated in this study are indicated in bold type. Coloured shading indicates the three networks of ionic interactions in the structure of sMMO detailed in the text. Among these, green indicates E320 (interacts with R12 in the β [MmoY] subunit of the enzyme). TomA3, toluene *ortho*-monooxygenase; PhN, phenol hydroxylase; TouA, toluene/*o*-xylene monooxygenase; TmoA, toluene-4-monooxygenase; OB3b-MmoX, MmoX from *Ms. trichosporium* OB3b (studied here); McBath-MmoX, MmoX from *Mc. capsulatus* (Bath); B272-AmoC, alkene monooxygenase from *Rhodococcus rhodochrous* B-276.

**Supplementary Table 1.** Sequences of oligonucleotides used for site-directed mutagenesis. P3 and P4, the forward and reverse external primers respectively, were used for construction of all mutants. Complementary pairs of mutagenic primers were used to create each mutation; the forward primer of each pair is shown. Mutations are shown in bold type. Codons encoding altered amino acids are underlined.

| Primer | Oligonucleotide sequence 5’ → 3’ |
| --- | --- |
| P3 | ATT CGA GCT CAA ACG TTC GAA C |
| P4 | GGG CTC TCG ACG CCA TAT TTG |
| R98L | TCC ATC CCC **T**CT GGG GCG AGA |
| F192I | TTC GCC GAC GGC **A**TC ATC TCC GG |
| I217A | AT CCG CTC **GC**C GTC GCC GTC AC |

benzyl alcohol

*p* cresol

100 nmoles 2-phenyl ethanol

toluene

**Supplementary Fig S2.** Representative chromatograph showing toluene and oxidation products benzyl alcohol and *p-*cresol following a 5 min incubation with cells of the native wild-type strain *Ms. trichosporium* OB3b. An internal standard of 100 nmol of 2-phenyl ethanol was added prior to solvent extraction

100 nmoles 2-phenyl ethanol

biphenyl

4hbp

3hbp

2hbp

**Supplementary Fig S3.** Representative chromatograph showing biphenyl and oxidation products 2-hydroxybiphenyl (2hbp), 3-hydroxybipheny (3hbp) and 4-hydroxybiphenyl (4-hbp) following a 1 h incubation with cells of recombinant *Ms. trichosporium* expressing the R98L mutant of sMMO. An internal standard of 100 nmol of 2-phenyl ethanol was added prior to solvent extraction.

**References**

Ho SN, Hunt HD, Horton RM *et al.* Site-directed mutagenesis by overlap extension using the polymerase chain-reaction. *Gene* 1989;**77**:51-9.

Smith TJ, Murrell JC. Mutagenesis of soluble methane monooxygenase. Methods Enzymol. 2011;**495**:135-147.
